# Expectations Matter: Social and Emotional Loneliness and Attitudes Toward Aging

**DOI:** 10.1093/geroni/igaf122.3582

**Published:** 2025-12-31

**Authors:** Abigail Hoffman, Kimberly Van Orden

**Affiliations:** University of Rochester, Fairport, New York, United States; University of Rochester Medical Center, Rochester, New York, United States

## Abstract

This study examines relationships between attitudes towards aging (internalized ageism) and loneliness using multidimensional measures. Few studies differentiate between types of loneliness or dimensions of internalized ageism, which is needed to develop targeted interventions. We hypothesized that greater global internalized ageism would be associated with both greater social and emotional loneliness because negative attitudes towards aging could impact an older person’s willingness to engage socially (resulting in social loneliness) as well as experiences of feeling connected to others (emotional loneliness). We also hypothesized that all three dimensions of internalized ageism (psychosocial loss, physical change and psychological growth) will be associated with both greater social and emotional loneliness. This is a secondary data analysis from the HOPE clinical trial (NCT03343483) that enrolled participants with clinically significant loneliness (n = 276; ages 60-92; 76.1% female; 90.6% white; 32.2% married) and who completed the 6-item DeJong Gierveld Loneliness Scale and the 24-item Attitudes to Ageing Questionnaire (AAQ). A multiple regression model with both forms of loneliness as simultaneous predictors of global internalized ageism indicated that both social (ß = -0.305, p<.001) and emotional loneliness (ß = -0.352, p<.001) were independently associated with internalized ageism (R2 = 0.321). Three regression models (for each dimension of internalized ageism) also indicated significant independent effects of both social and emotional loneliness on ageism dimensions. Limitations and future directions will be discussed, including planned analyses examining impacts of loneliness interventions provided in the HOPE RCT on dimensions of internalized ageism.

